# Giant Morgagni hernia and aorto-pulmonary collaterals in a Loeys–Dietz patient undergoing surgery for aortic root aneurysm and mitral valve prolapse

**DOI:** 10.1093/icvts/ivae136

**Published:** 2024-07-22

**Authors:** Federica Lo Presti, Giuseppe Palmiero, Giuseppe Limongelli, Alessandro Della Corte

**Affiliations:** Department of Translational Medical Sciences, Unit of Cardiac Surgery, Monaldi Hospital, University of Campania “L. Vanvitelli”, Naples, Italy; Inherited and Rare Cardiovascular Diseases, Department of Translational Medical Sciences, University of Campania “L. Vanvitelli”, Naples, Italy; Inherited and Rare Cardiovascular Diseases, Department of Translational Medical Sciences, University of Campania “L. Vanvitelli”, Naples, Italy; Department of Translational Medical Sciences, Unit of Cardiac Surgery, Monaldi Hospital, University of Campania “L. Vanvitelli”, Naples, Italy

**Keywords:** Loeys–Dietz syndrome, Morgagni hernia, aorto-pulmonary collateral arteries, aortic root aneurysm, mitral regurgitation

## Abstract

The case of a Loeys–Dietz syndrome patient undergoing mitral valve repair and composite aortic root and valve replacement is here described: preoperative CT scan unravelled a previously misdiagnosed Morgagni hernia (anterior diaphragmatic), containing omentum only, compressing the right ventricle. Intraoperatively, an abnormal oxygenated blood backflow into the left ventricle was observed, postoperatively found to be caused by major aorto-pulmonary collateral arteries. This is the 1st case of Morgagni hernia and systemic-pulmonary shunt ever reported associated with Loeys–Dietz syndrome. These congenital features may be important in both phenotyping and surgical management.

## INTRODUCTION

Loeys–Dietz syndrome (LDS) is a hereditary connective tissue disorder presenting with cardiovascular, facial, ocular, skeletal and joint manifestations. Systemic phenotype and disease severity vary depending on the underlying gene variant [1].

A case is here reported of elective aortic root and mitral surgery in a patient with LDS, with concomitant diaphragmatic anterior hernia previously mistaken for an intrathoracic lipoma, and anomalous systemic-to-pulmonary arterial communications suspected intra-operatively and ascertained only post-operatively.

## CASE PRESENTATION

A 34-year-old male was followed-up at the Rare Cardiovascular Diseases outpatient clinic of Monaldi Hospital (Naples, Italy) for LDS diagnosis: a targeted gene analysis with Sanger sequencing had demonstrated a TGFBR1 mutation 16 years before at the time of bilateral inguinal hernia correction. Systemic manifestations included tall stature, straight back, craniosynostosis, bifid uvula, arachnodactyly, ‘pectus carinatum’ and wide scars.

**Figure 1:**
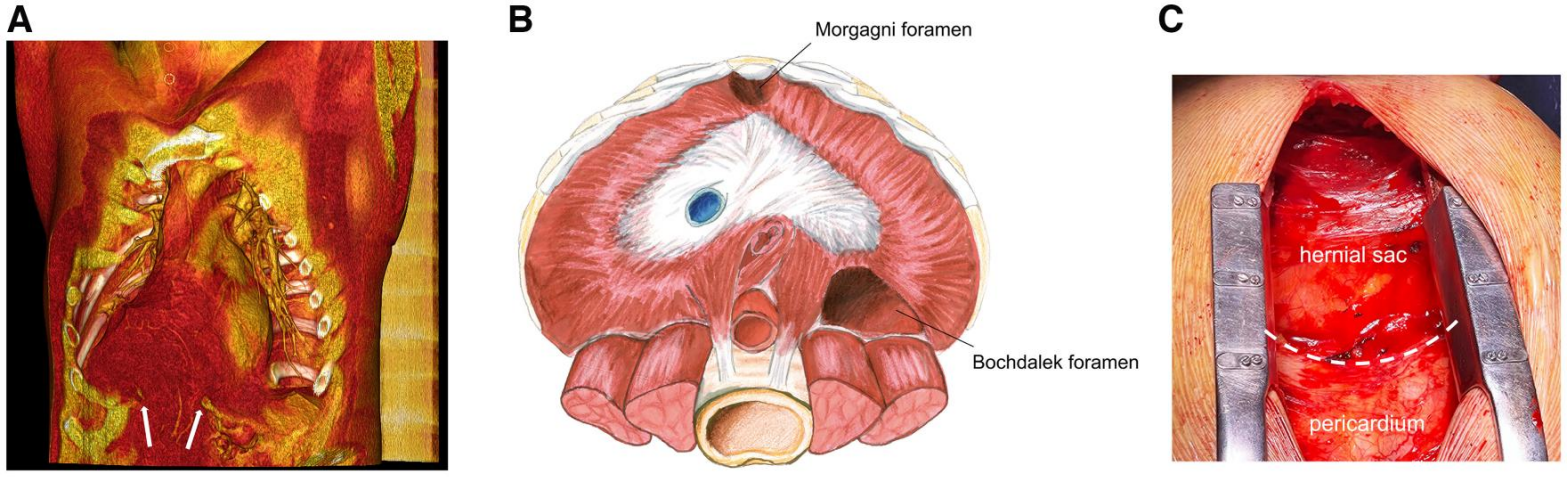
(**A**) Pre-operative angio-CT scan: three-dimensional rendering of the hernial sac (red, anteriorly to the pericardium, yellow). White arrows indicate the edges of the diaphragmatic defect; (**B**) anatomical drawing showing Bochdalek and Morgagni ‘foramina’ (re-drawn and modified from: Sanford et al.) [6]; (**C**) intraoperative photograph after median sternotomy (patient’s head at the bottom): the white dashed line indicates the boundary between hernial sac and pericardium.

When follow-up echocardiography showed severe mitral regurgitation due to posterior leaflet prolapse and aortic root dilatation, with moderate aortic regurgitation and dilated left ventricle, the patient was referred to the Cardiac Surgery Unit of our Hospital. Angio-CT scan documented an asymmetric 49-mm root aneurysm with elongated ascending aorta. A collateral finding was a voluminous Morgagni hernia, misdiagnosed in previous hospital admissions as a lipoma: a 13 × 11 × 5 cm omentum sac, dislocated in the anterior mediastinum through a 6-cm wide hernial orifice in the diaphragm, significantly compressing the right ventricle (Fig. 1). In multidisciplinary team, the consensus was to defer laparoscopic repair of the hernia after the cardiac procedure: direct suture was excluded because of the orifice width, whereas the abdominal approach was considered the most suitable option for mesh positioning, in terms of long-term results.

At operation, after median sternotomy, the hernial sac (Fig. 1) was cautiously dissected from the pericardium; stay sutures on the pericardiotomy edges kept the *omentum* out of the operative field throughout the procedure. Cardiopulmonary bypass (CPB) was instituted through aortic and bi-caval cannulation, a vent line inserted in the right upper pulmonary vein, and Del Nido cardioplegia administered into the coronary ostia. Extensive para-commissural fenestrations of the aortic cusps prompted the choice to replace rather than repair the valve.

Notwithstanding total cardiopulmonary bypass, a significant amount of arterial blood filled the left ventricle, overcoming the vent capacity and hampering visualization of the aortic valve and root. No patent ‘ductus arteriosus’ was found: hypothesizing other systemic-to-pulmonary communications, the right and left pulmonary arteries were isolated and cross-clamped. From then on, surgery was accomplished smoothly, in a bloodless operative field.

Through left atriotomy, the mitral valve was repaired by P2 triangular resection and posterior annuloplasty. A Bentall procedure was performed using an On-X valved Valsalva prosthesis (valve size 25 mm; graft size 26 mm).

**Figure 2:**
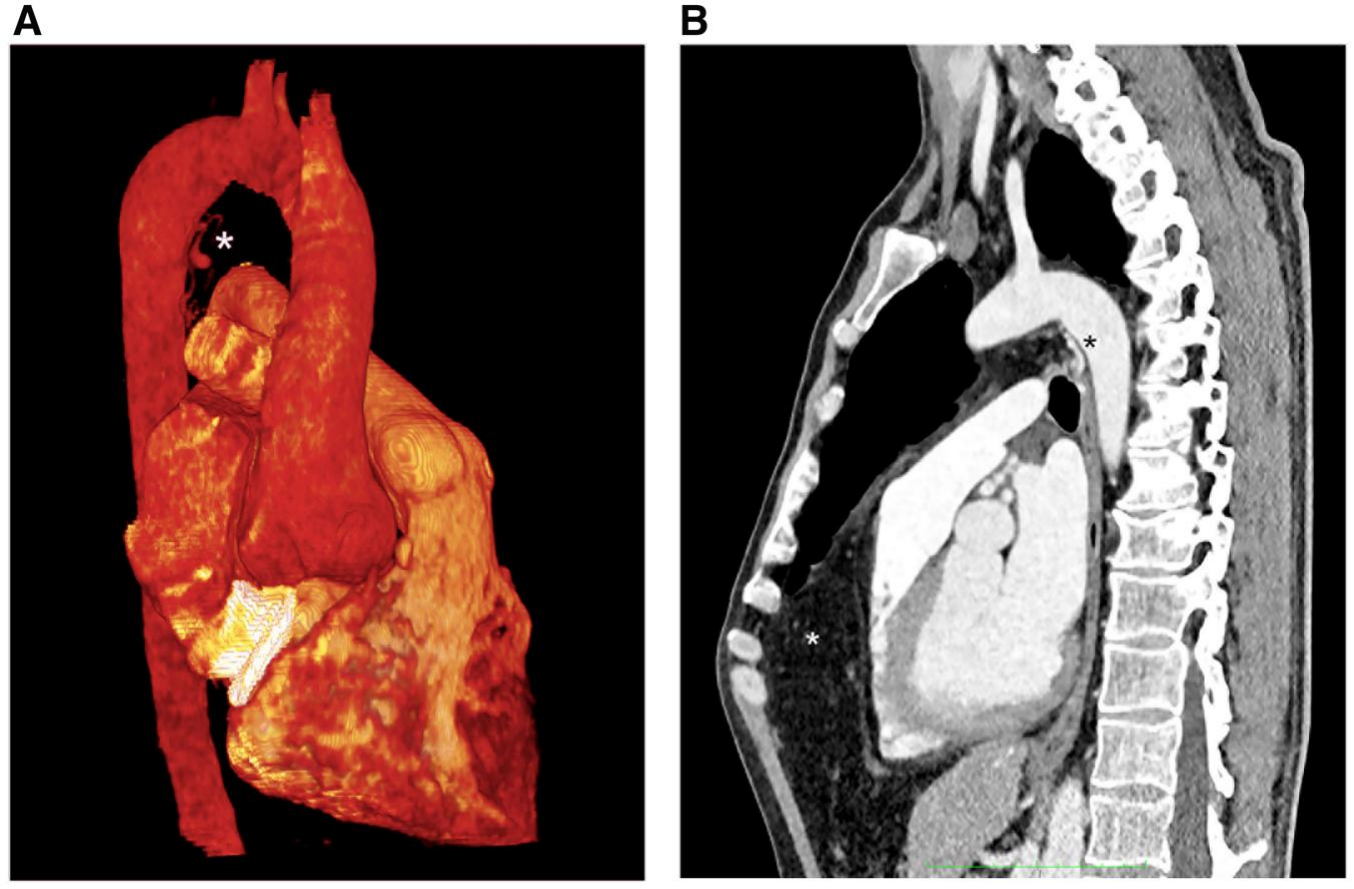
(**A**) Collaterals leading from the anterior aspect of the proximal descending aorta into the pulmonary arteries (white asterisk); (**B**) sagittal view showing coexistence of Morgagni hernia (white asterisk) and MAPCAs (black asterisk). MAPCAs: major aortopulmonary collateral arteries.

Postoperatively, angio-CT scan was reviewed, and major aortopulmonary collateral arteries (MAPCAs) were identified raising from the anterior aspect of the proximal descending aorta and distributing to the main branches of the pulmonary arteries bilaterally (Fig. 2). Postoperative course was uneventful and the patient was discharged home on 8th postoperative day. Six months later, laparoscopic hernia repair was successfully performed.

## DISCUSSION

The case here reported is an example of severe LDS phenotype, in which cardio-aortic involvement was associated with multiple systemic features, including diaphragmatic and vascular malformations.

Morgagni hernias are extremely rare in adulthood. Failure of insertion of the anterior diaphragm to the costal arches during embryogenesis implies Morgagni’s ‘foramen’ persistence (Fig. 1B), behind the xiphoid. When the ‘omentum’ is the herniating organ (75% cases), patients are generally asymptomatic and diagnosis is formulated incidentally by imaging [2]. There are only few reports of congenital diaphragmatic hernias in LDS patients, however, of the more frequent Bochdalek type (postero-lateral; Fig. 1B) [3, 4]. In the present case, the hernia had been previously interpreted as a lipoma, overlooking the diaphragmatic defect.

MAPCAs consist of non-regressed systemic-to-pulmonary embryologic connections from the aorta or its branches to the pulmonary arterial vasculature: they can compensate for pulmonary low-flow in congenital cyanogenic defects. As this was not the case, we can hypothesize the responsibility of transforming growth facotr-beta (TGF-β) pathway alterations, with impairment of vasculogenesis [5], for the MAPCA in our patient, possibly also maintained by reduced pulmonary flow due to compression by the hernia on the right ventricle.

The present case report suggests awareness of the possible presence of diaphragmatic hernias and MAPCAs in connective tissue disorder patients (often needing thoracic or cardio-aortic surgery). During operation, clamping the 2 main pulmonary artery branches stopped or critically hampered the flow in the anomalous connections without complications. A correct preoperative diagnosis of the MAPCAs would have allowed their interventional embolization before surgery or suggested an intraoperative temporary occlusion of the pulmonary veins, with pulmonary artery venting.

## Data Availability

Clinical data here presented are available for direct review upon reasonable request.
